# Found in the Folds: A Rediscovery of Ancient Egyptian Pleated Textiles and the Analysis of Carbohydrate Coatings

**DOI:** 10.3390/molecules27134103

**Published:** 2022-06-25

**Authors:** Jennifer Poulin, Chris Paulocik, Margaret-Ashley Veall

**Affiliations:** 1 Canadian Conservation Institute, 1030 Innes Rd., Ottawa, ON K1B 4S7, Canada; margaret.veall@pch.gc.ca; 2 Royal Ontario Museum, 100 Queen’s Park, Toronto, ON M5S 2C6, Canada; chrisp@rom.on.ca

**Keywords:** Ancient Egypt, pleated linen textiles, polysaccharides, starch, gum arabic, fruit gum, gas chromatography-mass spectrometry, pyrolysis-gas chromatography-mass spectrometry, microscopy

## Abstract

Charles T. Currelly, first director of the Royal Ontario Museum, participated in excavations of the tomb of King Nebhepetre, now known as Mentuhotep II, (Dynasty XI) in Deir el-Bahri, Egypt in 1906. He brought to Canada many objects from the excavations, and objects that he purchased while in Egypt; these formed the initial collection of the museum. Among the objects were seven fragments of fine linen cloth with intricate pleat patterns. Recently, the cloths became the subject of a study to learn how they had retained their pleats for 4000 years. Samples were examined and analysed using polarised light microscopy, scanning electron microscopy-electron dispersive X-ray spectrometry, gas chromatography-mass spectrometry, and pyrolysis-gas chromatography-mass spectrometry. Three of the cloths were likely fragments of clothing re-purposed as bandages and were found to be saturated in mummification balms composed of *Pinaceae* resin, *Pistacia* resin, and an essential oil characterised by a high abundance of cedrol, possibly originating from a juniper species. All seven of the cloths were found to have traces of polysaccharides from two probable sources: an arabinogalactan gum such as gum arabic or a fruit gum, and a polyglucoside, possibly starch.

## 1. Introduction

Collections may be considered a museum’s most valuable asset in terms of their ability to shape strategies and values, as a source to create experiences to engage and attract visitors, to be a physical archive for research among professionals within the heritage sector and, in some cases, may be a source of income to perpetuate the longevity of the museum spaces themselves. The re-discovery of objects is not an uncommon phenomenon within museum collections stores, and both the heritage experts and popular media alike periodically embrace these stories of rediscovery as opportunities to re-evaluate existing paradigms and re-ask questions that in the past were generally thought to be unanswerable.

The first Director of the Royal Ontario Museum (ROM), Charles T. Currelly, had a talent for collecting and dedicated his life to the development of the ROM. Prior to his appointment as director, Currelly excavated in Egypt (1905–1907) and also purchased many objects that formed the initial collection of the ROM. His publication, *I Brought the Ages Home,* outlines his archaeological field work with Flinders Petrie and his quest to establish a museum as part of the University of Toronto (which would ultimately become the ROM) [1].

**Figure 1:**
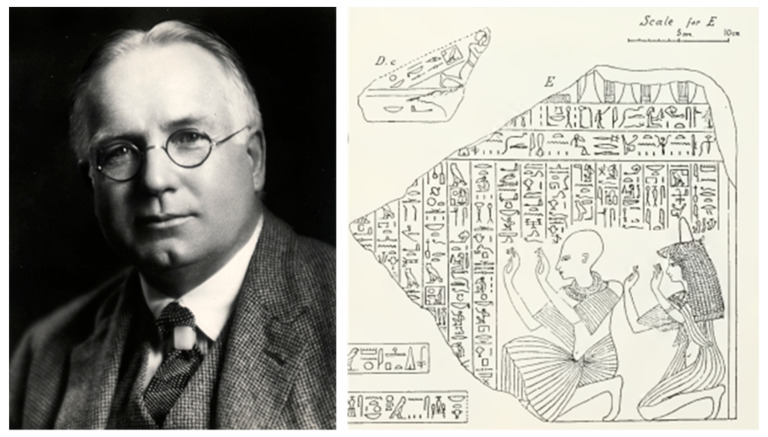
Charles T. Currelly, first Director of the Royal Ontario Museum; Attribution: University of Toronto Archives, 2004-30-7MS, public domain. Line drawing made by Currelly during excavation of the tomb of King Nebhepetre, also known as Mentuhotep II, (Dynasty XI) in Deir el-Bahri, Egypt in 1906 [2], Plate VIII, public domain.

The purpose of the analysis highlighted in this paper was precipitated by the examination of a large group of archaeological textile fragments from the ROM’s Egyptian collection in preparation for gallery rotations, focused on selecting enough artefacts to carry out rotations of light sensitive textile material periodically for several years. An investigation of the many drawers of fragments in the Egyptian department uncovered an impressive range of artefacts, including pleated, beaded, painted, and inscribed textiles. According to the card files, most of the fragments had been acquired by Currelly in Egypt at Deir el-Bahri (Figure 1). It is known that he was a participant in the excavations at this site and possibly a leader during the operation [1,2,3]. The tomb had been plundered prior to this excavation, and an account of the findings remarked upon the “heaps of mummy cloths” that were left behind [1]. These fragments of interest are from the excavation carried out on the tomb of King Nebhepetre, now known as Mentuhotep II (Dynasty XI, 2060–2009 BCE) at Deir el-Bahri, Egypt. Early excavations at the site proved to be very successful in uncovering a variety of objects. Indeed, in a letter from Currelly to a Mr. Walker from the excavation at Deir el-Bahri on 8 December 1905, Currelly writes, “[w]e are having wonderful success, things are simply tumbling out of the mounds, sculpture, paintings on linen, enamels and tools mostly, but other stuff as well” [4].

At the ROM, over one hundred years after the original accession of the items, the fragments that were of particular interest were a collection of finely pleated linen textiles that had retained their crisp pleats, intact after several thousand years. Tomb illustrations, an example of which is drawn by Currelly’s hand and shown in Figure 1, are numerous, depicting men wearing pleated kilts or women in pleated dresses [2]. These items must have been popular in Egypt’s hot climate, since the pleating gave greater volume with the additional material allowing for greater airflow and freedom of movement resulting in more flexibility for the wearer. 

Examples of fragments in the drawers ranged from single pleated textiles to a more complicated double pleat with folds carried out both horizontally and vertically; indeed, some of the examples had pleating intervals of 16 mm. This finer linen fabric was of a higher quality and probably easier to pleat. In order to create a pleat, it is necessary to double the fabric over itself thereby using more fabric yardage. Due to the uniformity and crispness of the pleats among the Egyptian examples, it is surmised that some sort of pleating board or device was utilized to achieve that quality of pleat [5,6,7,8]. It also seems likely that it would have been necessary to have the garments re-pleated after use. The use of a fine linen fabric, using an excessive amount of yardage, and the likelihood of repeated pleating after use, suggests that these textiles would have been considered a luxury item reflecting wealth and power. Pleated garments were often depicted in contemporaneous statuary and stone carvings as very stiff and protruding from the body, and sometimes in darker tones than the non-pleated portions of their garments. It has been postulated that they may have been starched or similarly stiffened; however, no such reinforcement has ever been identified [5,6].

In this paper we present a combined methodology featuring microscopy, scanning electron microscopy/ energy dispersive X-ray spectrometry (SEM/EDS), and two chromatography-mass spectrometry techniques to identify the types of commodities that may have been employed as coating and stiffening agents to achieve this permanent stiffness. In this we aim to highlight a multi-method approach to identify mixed organic commodities utilized in Ancient Egyptian contexts. In doing so, we address the complexity of identifying carbohydrate-derived materials from ancient contexts along with demonstrating the value of revisiting items curated from collections long ago to shed light on quotidian and ritual aspects of life in ancient cultures.

## 2. Results and Discussion

### 2.1. Microscopic Examination

**Figure 2:**
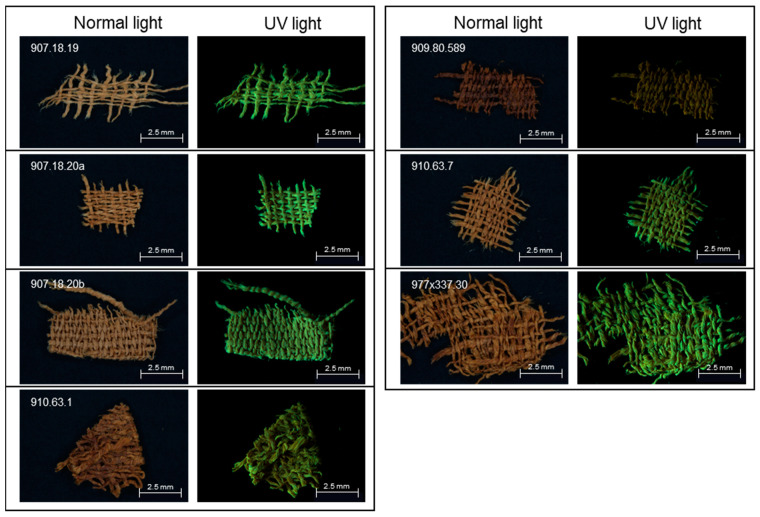
Photomicrographs of small samples from the seven linen fragments. The images were acquired under normal and UV light illumination.

Photomicrographs of small fragments from the seven linen objects are shown under normal and raking UV light illumination in Figure 2. Each cloth was woven in uneven 1/1 plain weave; however, since none of the fragments has a selvedge, it is not known which are warp-faced or weft-faced; both types have been identified in Ancient Egyptian linen fabrics [9]. Except for object 977 × 337.30, which is very heavily saturated with dark resinous material, all fragments show inhomogeneous bright green fluorescence under UV illumination. Evident dark resinous coatings are also present on the fragments from cloths 907.18.20a and 907.18.20b, yet they fluoresce much more brightly than 977 × 337.30, which may indicate that the resin mixture was applied more thickly to this latter cloth. It is possible that the three darker cloths originate from mummy wrappings, as resin-soaked cloths were commonly laid over the body after embalming.

The question of how the pleats were set into the individual cloths lead to the premise that they may have been coated with starch, just as one might treat a modern-day cloth intended to be pleated. Examination of the intact threads by polarised light microscopy (PLM) did not identify any intact starch granules. The threads were also examined under a stereomicroscope and stained with potassium iodide/iodine (Lugol’s stain). Although some of the stained threads did show amorphous particles of black colouring along the surfaces of the threads, which may indicate a possible presence of gelatinised starch, the lack of continuity and the difficulty in visualizing the staining along the surface, particularly given that a coating would be expected to have a relatively continuous distribution, rendered this stream of examination inconclusive. Photomicrographs of the threads before and after staining can be found in the Supplementary Materials (Figure S1).

### 2.2. Instrumental Analysis

#### 2.2.1. Elements: Scanning Electron Microscopy/Energy Dispersive X-ray Spectrometry

Elemental analysis by SEM/EDS was undertaken to determine the possible presence of fibre-bound mineral dyes, such as ferrihydrite (iron buff), which has been reported on Egyptian linen textiles, both from Ancient Dynastic Egypt [10] and the more recent Byzantine and early Medieval periods (4–9th centuries CE) [11]. All threads were found to consist mainly of carbon and oxygen, typical for cellulosic fibres, and common traces of silicon and calcium were also noted for the majority of the fibres; this may be attributed to minerals from the depositional environment. The cloth (977 × 337.30) that appeared darkest under UV light (Figure 2) and that has the thickest coating of dark brown embalming resin was also found to contain traces of sodium, possibly from contact with natron, a commonly used desiccant during mummification comprised of a mixture of sodium carbonate decahydrate, sodium bicarbonate, sodium sulfate and sodium chloride [12]. This cloth also presented a trace abundance of elemental iron which was determined to be present as a likely contaminant from the burial tomb, and not in a fibre-bound state.

#### 2.2.2. Non-Polymeric Organic Components: Gas Chromatography-Mass Spectrometry

**Figure 3:**
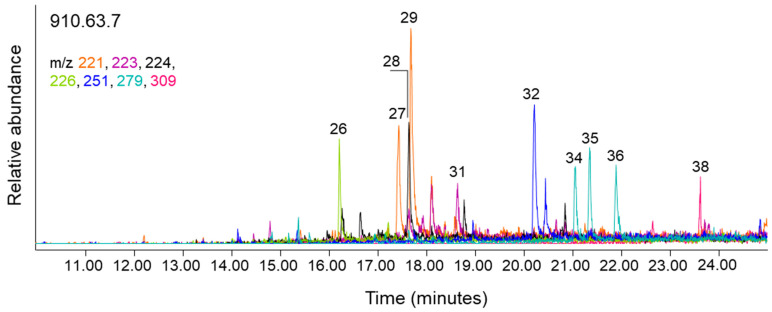
Extracted ion chromatogram (EIC) created using *m*/*z* 221, 223, 224, 226, 251, 279, and 309 for cloth 910.63.7. Peak labels correspond to compounds listed in Table 1.

The linen textiles visually appear to be shades of yellow-brown to dark brown, seemingly due to degradative processes that occur as cellulose ages and oxidises [13,14,15]. Additionally, for three of the cloths, the presence of dark brown resinous material contributes to their appearance. Previous studies, however, have identified organic or mineral dyes such as safflower or iron buff on Ancient Egyptian linen textiles [10,16,17], and there are extensive case studies on the identification of the botanical resources utilised in embalming rituals throughout the Ancient Egyptian chronologies [18,19,20,21,22,23,24]. With the potential of multiple sources of organic materials, including the possibility of intact or degraded organic dyes that may be present on the cloths, threads from each textile were extracted in m-(trifluoromethyl)phenyltrimethylammonium hydroxide (TMTFTH) and toluene and subsequently analysed using GC-MS [25]. This technique is useful for investigating non-polymerised organic components from textiles. With the exception of textile 909.80.589, which was a smaller sample and yielded a weak extract, the extracts from each of the thread samples contained compounds related to hydrolysable tannins and humic substances [26,27,28,29]. To illustrate these results, an extracted ion chromatogram (EIC) from textile 910.63.7 (*m*/*z* 221, 223, 224, 226, 251, 279, and 309) is shown in Figure 3; the remaining EICs are presented in the Supporting Information (Figure S2). The extracted peaks show many of the compounds related to hydrolysable tannins and humic substances that are present on the cloths, including methylated derivatives of gallic acid (26), benzenetricarboxylic acids (27 and 29), a hydroxybenzenedicarboxylic acid (31), a hydroxybenzenetricarboxylic acid (32), benzenetetracarboxylic acids (34, 35, and 36), and a hydroxybenzenetetracarboxylic acid (38). All of the benzenecarboxylic acid compounds shown in Figure 3 are typical for textiles that have been exposed to tannin-containing plant material. This contact can occur through dyeing or mordanting, and also through exposure to water from a tannin-rich source during cleaning or processing of the cloth or fibres. Exposure can also occur at wet bog burial sites [30]. It is also possible that some of the tannins and humic substances present in these samples may originate from exposure of the fibres to degrading plant material during the fermentation process of retting the flax plants [7]. If this is the source, it would be reasonable for all of the cloths to contain similar compounds. Notably, there is no evidence in these extracts of compounds that might arise from the degradation and oxidation of lignin, which is present at approximately 2% in retted linen fibres [31]. Because the lignin polymer in linen is predominantly composed of guaiacol units [32], compounds including fully methylated guaiacol (1,2-dimethoxybenzene) and 4-vinyl guaiacol (1,2-dimethoxy-4-vinylbenzene) in the chromatograms would have been an indication of the degradation process [32], and these were not detected. It is assured that any lignin present in these cloths has undergone some degradation; however, the indicator compounds may not be detected due to very low lignin content, as might be the case for fine, white linen cloth.

**Figure 4:**
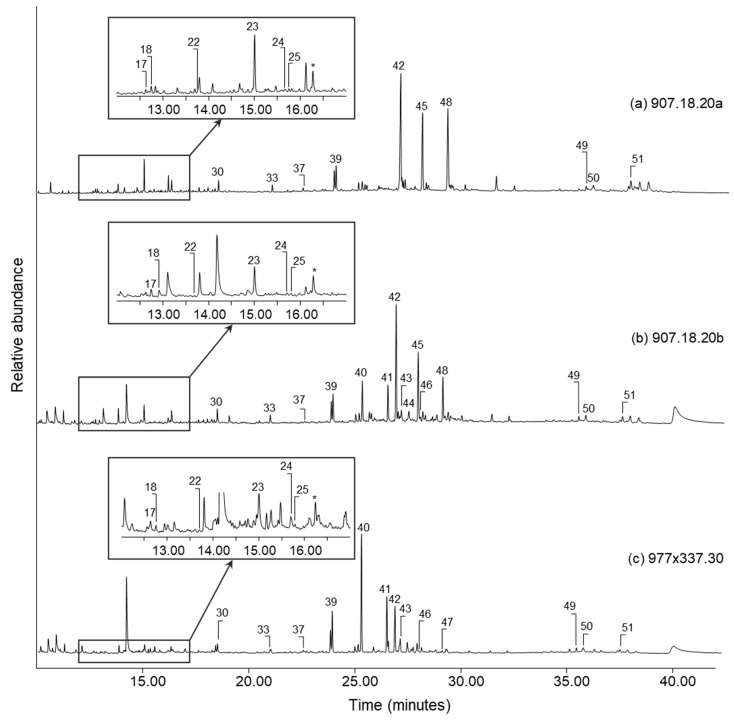
Total ion chromatograms from cloths with visible embalming resins (907.18.20a, 907.18.20b, and 977 × 337.30). A product derived from the pesticide pentachlorophenol is labelled with an asterisk (*). Peak labels correspond to compounds listed in Table 1.

The TMTFTH extracts from the three cloths with visible resinous coatings (909.18.20a, 909.18.20b, and 977 × 337.30) provided the most complex chromatograms indicative of organic mixtures. Total ion chromatograms (TICs) of these extracts are shown in Figure 4. In the three chromatograms, one of the key constituents was identified as highly oxidized *Pinaceae* resin. Compounds attributed to this component include methyl esters of dehydroabietic acid (39), 7-methoxy-tetradehydroabietic acid (40), and 7-oxo-dehydroabietic acid (42) [33]. Additional peaks indicating the advanced state of oxidation of the resin on each cloth are also present and include methyl esters of 7-methyoxy-pentadehydroabietic acid (41), 7-oxo-tetradehydroabietic acid (45), and 15-hydroxy-7-oxo-dehydroabietic acid (47) [33]. Due to ageing, oxidation and loss of species-identifying markers, the conifer type(s) was not determined [34]. The presence of trace relative abundances of retene in the TICs may indicate that the *Pinaceae* resin in the balm coatings was thermally processed, at least mildly [35,36]. However, the very low abundance of the retene relative to the other diterpenoid compounds does not seem to indicate the presence of strongly heated *Pinaceae* tar. Conifer resins are commonly found in Egyptian mummification balms [21,22,37,38,39,40].

A series of small peaks eluting late in the TICs indicate the presence of a triterpenoid resin in the coatings on each of the three cloths in Figure 4. The peaks include three unidentified compounds (48, 49, and 50) having characteristic mass ion fragments *m*/*z* 189, 203, and 262, which are typical for lupane, ursane and oleanane compounds [41], respectively. The additional presence of moronic acid (3-oxo-olean-18-en-28-oic acid, 51) likely indicates that the resin is mastic, derived from *Pistacia* genus trees [42,43]. These trees grew in the Mediterranean at the time that the cloths were created [44]. Like conifer resin, mastic resin is a commonly identified ingredient in Egyptian mummification balms [21,22,37,38,39,40]. In previous studies, nororlean-17-en-3-one has been designated as a marker for heated *Pistacia* resins [44,45]. The absence of this marker in the chromatograms in Figure 4 may indicate that the mastic resin was not strongly heat-processed in the manufacture of the balm used on the three cloths.

Sesquiterpene compounds are also present in the resinous balms on the three cloths. Details of the chromatograms have been expanded in Figure 4 to highlight these peaks. The most abundant sesquiterpene detected in each chromatogram is cedrol (23), and smaller peaks of α-cedrene (17), β-cedrene (18), cuparene (22), calamenene (24), and cadalene (25) have also been identified. The latter three compounds are associated with *Pistacia* resin [46]; therefore, this must account for at least a portion of the amounts detected in the resin coatings. More significant, however, is the possibility that the majority of the sesquiterpene compounds identified on the cloths may originate from juniper oil (such as *Juniperus oxycedrus*), or a mixture of conifer oils of which juniper is a major constituent [18,47,48]. This conclusion is based on the high relative abundance of cedrol and the additional presence of α-cedrene and β-cedrene, which is consistent with juniper oil [48]. The purpose of including essential oils from conifer sources in mummification balms was possibly to stave off microbial attack and help conserve biological tissue [48].

Very low abundances of monocarboxylic fatty acids, mainly palmitic acid (C16:0, 30) and stearic acid (C18:0, 33), are present in the three cloths shown in Figure 4, and also on the four cloths without visible coatings. It is difficult to assign such peaks to a source, given the pervasiveness of these compounds in nature. They might derive from any of the plant-sourced resin components on the cloths or originate from a separately added component such as a plant or animal oil [21,22,37,38,39,40].

One out of place component is present in all three chromatograms in Figure 4. 2,3,5,6-Tetrachloroquinone is the key degradation product of the pesticide pentachlorophenol [49], and the presence of the methylated derivative (*) in these extracts may indicate that this pesticide was used at the ROM in a past treatment.

The combination of *Pinaceae* resin, mastic resin, and a conifer essential oil (probably sourced from a juniper tree or shrub) is consistent with embalming mixtures that have been previously characterised in Ancient Egyptian mummies, mummy wrappings, and funerary jars [21,22,38,39,50]. Mummy bandages were often made from old clothing [7], and it is notable that although resin-saturated, these three cloth fragments have retained a pleated appearance. In his memoir, Currelly briefly describes the types of textiles that they encountered during the excavations at Deir el-Bahri. He differentiates between those that were used as mummy wrappings and articles of clothing [1]: “In front of the temple the king had buried six of his wives, one of who, Henhenet, Hall had found, stripped naked by robbers hunting for her jewellery, lying on a pile of her wrappings and two of her shawls.” Although Currelly does not provide any further descriptions of the cloths in the memoir, it is probable that he could differentiate based on the obvious presence of dark brown coating on the wrappings, and the extra inflexibility that this gave to the cloths.

#### 2.2.3. Threads and Coatings: Pyrolysis-Gas Chromatography-Mass Spectrometry

**Figure 5:**
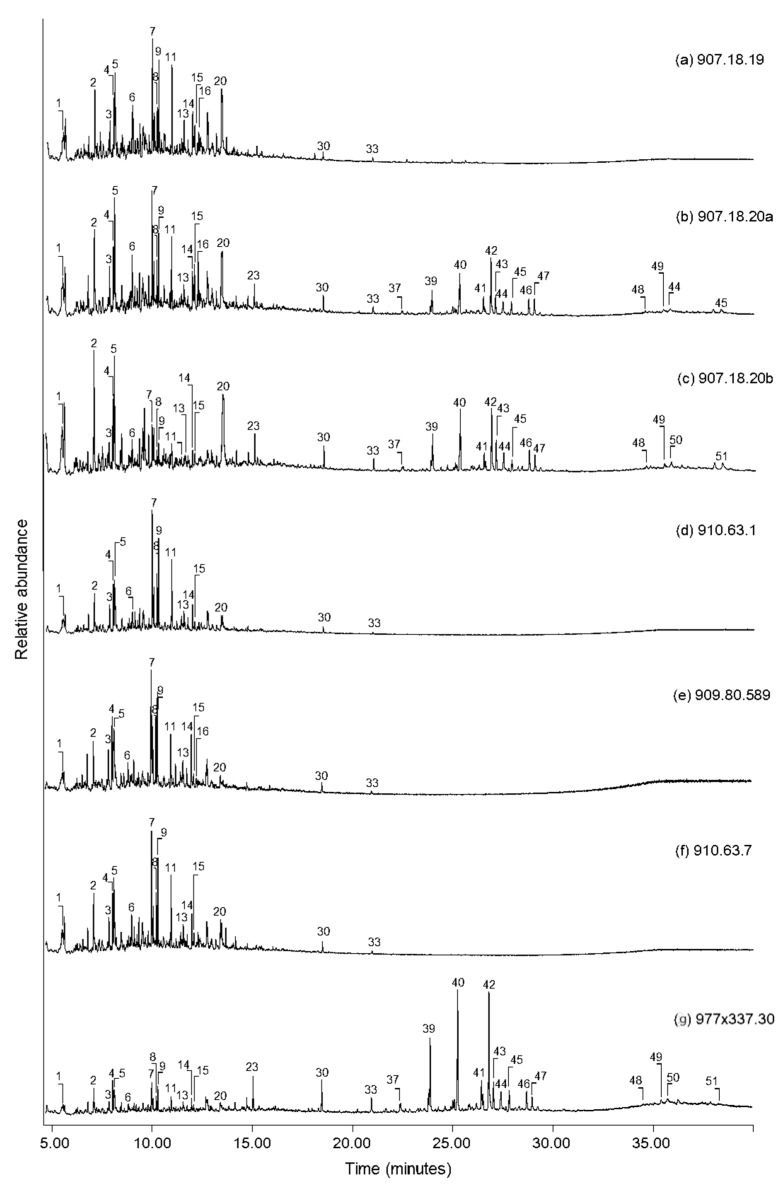
Total ion chromatograms obtained by TMAH Py-GC-MS of threads from pleated linen textiles. Peak labels correspond to compounds listed in Table 1.

Consistent with linen, Py-GC-MS analysis with tetramethylammonium hydroxide (TMAH) derivatisation of whole threads from the textiles showed that they each contain compounds originating from the pyrolysis and methylation of cellulose. Additionally, the three textiles having embalming coatings also show abundant peaks from *Pinaceae* resin components and smaller peaks of triterpenoids from mastic resin which were previously described from the TMTFTH extractions and GC-MS analysis (Section 2.2.2). TICs from each textile are presented in Figure 5, and the most abundant components are labelled on the chromatograms and described in Table 1. Compounds in the TICs that derived from the linen fibres include furans (1,7), cyclopentenones (2,3), hydroxybenzenes (4, 6, 9, 11, 13, 14), and the anhydro sugar levoglucosan (1,6-anhydro-β-D-glucopyranose, 20) [51]. Of the several unidentified cellulose-derived compounds present, a notable marker that forms through TMAH derivatisation with Py-GC-MS is compound 15, having the characteristic mass spectral ions *m*/*z* 88, 73, 103, 135. Another peak (compound 16) eluting in each chromatogram just after this cellulose marker is also unidentified and presents an interpretation challenge. Compound 16 has the characteristic mass spectral ion fragments *m*/*z* 101, 99, 127, 71, and 75. It remains unknown after being first reported by Fabbri and Helleur more than 20 years ago [51]. When first discovered, it was described as a degradation product formed through TMAH thermochemolysis reactions of both cellulose and starch, possibly the dehydrated and partially methylated product of a deoxy-hydroxymethyl-pentonic acid [51]. Although acknowledged to be present in low relative abundances in cellulose [51], it is present in such a high abundance in the TMAH pyrolysis of starch that it has been suggested as a starch marker [51,52]. In a more recent study, Schilling et al. referred to this unknown compound as Schellmannose [52], and it has been published under this name multiple times [52,53,54,55,56]. In studies conducted at the Canadian Conservation Institute (CCI), very low relative abundances of this compound have been found to form during TMAH Py-GC-MS analysis of other materials containing glucose linkages, including dextrin and sucrose. However, methylated derivatives of sucrose can be identified through TMAH Py-GC-MS, and since these compounds were not detected on the threads, sucrose is not the source of Schellmannose (16) in this study.

**Figure 6:**
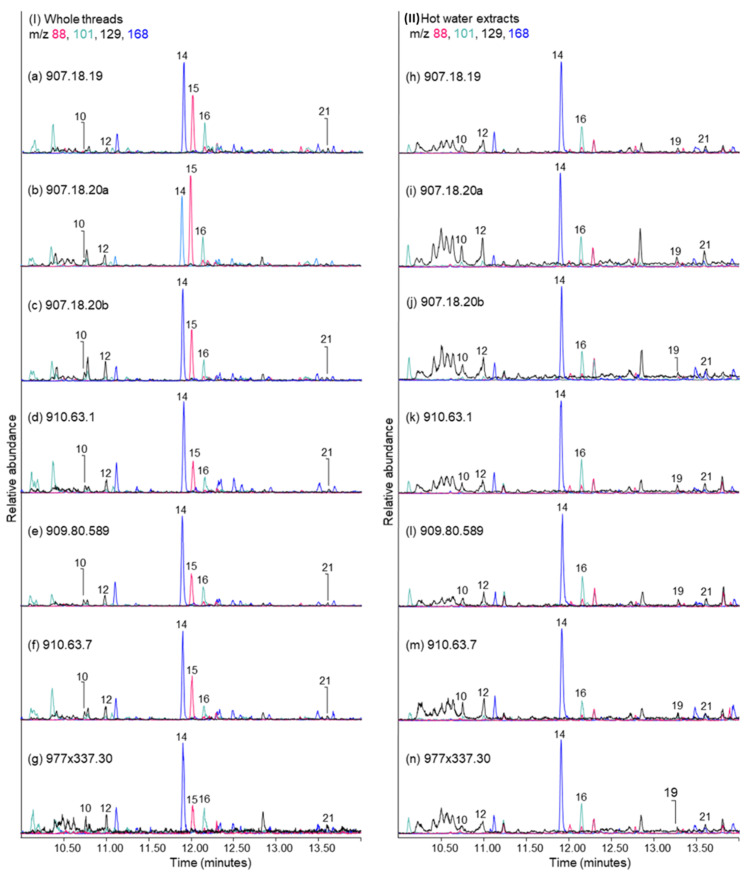
Partial extracted ion chromatograms (*m*/*z* 88, 101, 129, 168) obtained by TMAH Py-GC-MS for whole threads from the pleated linen textiles (**left**), and for the hot water extracts of the threads (**right**). Peak labels correspond to compounds listed in Table 1.

Further investigation of the unknown Schellmannose compound (16) and its origin on the pleated textiles, whether from cellulose or starch, was undertaken. Because starch is soluble in warm water and cellulose is not, small threads from each textile were extracted in hot (75 °C) deionised water and the extracts were dried and analysed using TMAH Py-GC-MS. Figure 6 shows side-by-side EICs obtained from the TMAH pyrolysis of the whole threads (left side of Figure 6) and the hot water extracts of the threads (right side of Figure 6). For the ion extractions, *m*/*z* values of 88, 101, 129, and 168 were used. The *m*/*z* 88 EIC highlights the presence of compound 15, the unidentified marker for cellulose; *m*/*z* 101 was chosen to show the presence of Schellmannose [51]; *m*/*z* 129 highlights methylated 3-deoxy-aldonic acid pyrolysates that form during TMAH pyrolysis of plant gums [57], and *m*/*z* 168 was chosen for 1,2,4-trimethoxybenzene, a pyrolysate common to many carbohydrates, including starch and plant gums [58]. In the EICs obtained from the whole thread analyses, three main peaks are present for each textile: the saccharide marker (1,2,4-trimethoxybenzene, 14), the unidentified cellulose marker (15), and Schellmannose (16). In addition to these compounds, small peaks of methylated 3-deoxy-D-*threo*-pentonic acid (10) and 3-deoxy-D-*erythro*-pentonic acid (12) are also present in each chromatogram. These compounds form during TMAH thermochemolysis reactions of polysaccharides containing arabinose and/or xylose [57,58].

One of the key differences between the EICs obtained from the analysis of the hot water extracts and those from analysis of the whole threads (Figure 6) is that the cellulose marker peak (15) is missing from the hot water extracts. This is attributed to the extracts only containing water-soluble components from the threads, while the whole thread chromatograms contain all those components in addition to the insoluble cellulose substrate as well as any other insoluble components. This is also important considering the Schellmannose question, and whether it is originating on these threads from the cellulose component of the linen or from a starch-containing additive. Unlike the cellulose marker (15), the Schellmannose peak (16) is present in each water extract, and this indicates that at least a portion of the unknown Schellmannose compound originates from a source that is water-soluble.

**Figure 7:**
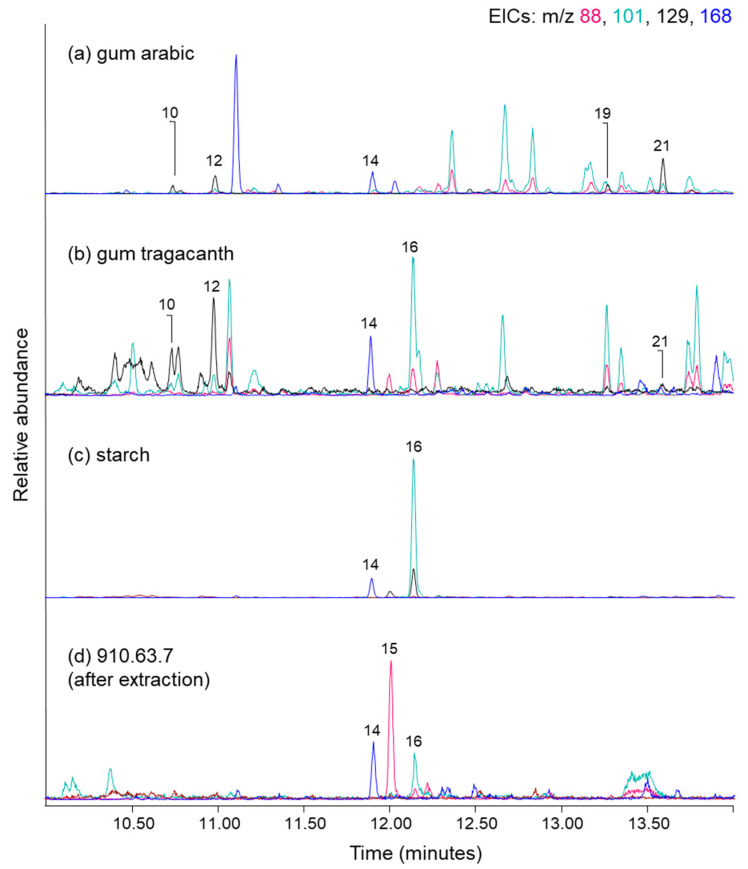
Partial extracted ion chromatograms (*m*/*z* 88, 101, 129, 168) obtained by TMAH Py-GC-MS for hot water extracts of commercial reference materials including (**a**) gum arabic, (**b**) gum tragcanth and (**c**) starch, and (**d**) the ancient cellulosic thread (910.63.7) after it had been subjected to hot water extraction. Peak labels correspond to compounds listed in Table 1.

Other differences between the whole thread and the water extract chromatograms in Figure 6 include an increase in relative abundances of the arabinose/xylose pyrolysates (10, 12), and the more evident presence of methylated 3-deoxy-D-*xylo*-hexonic acid (19) and 3-deoxy-D-*lyxo*-hexonic acid (21). These latter two compounds are TMAH pyrolysates that form from polysaccharide chains containing galactose units [57,58]. The presence of both arabinose or xylose, and galactose markers from a polysaccharide source indicates the presence of a plant gum, such as a fruit gum, gum tragacanth (*Astragalus* genus) or gum arabic (the acacias, incl. *Vachellia nilotica* and *Senegalia senegal*), all of which have been identified through gas chromatographic analysis of objects from Ancient Egypt, including paint binders, coatings, textile adhesives and cosmetics [58,59,60,61,62,63,64,65]. To aid in the identification of the gum, and any other polysaccharide that might be present, a variety of reference materials were subjected to hot water and the dried residues were analysed by Py-GC-MS with TMAH derivatisation. Figure 7a–c show the combined EICs for commercial gum arabic, commercial gum tragacanth, and starch, respectively. Also included in Figure 7 are the results from the analysis of ancient linen. Figure 7d shows the combined EICs for fibres from cloth 910.63.7, which were analysed after they had been extracted in hot water. The marker peaks present in the gum arabic EICs include pyrolysates of polymerised arabinose/xylose (10, 12) and galactose (19, 21), and saccharide marker 1,2,4-trimethoxybenzene (14). Peaks present in the gum tragacanth EICs include arabinose/xylose markers (10, 12), the saccharide marker (14), and, due to the presence of some starch in gum tragacanth’s composition [66], the unknown Schellmannose peak (16) is also present. Although the gum tragacanth polysaccharide chain does contain a minor abundance of galactose (approximately 10%), only the most abundant pyrolysate marker (19) from the pair is discernible in Figure 7b. The EICs constructed for the hot water extract of the starch contain a relatively minor abundance of the ubiquitous saccharide marker (14), but Figure 7c is dominated by Schellmannose (16). It is not difficult to see why this compound was designated as a starch marker by Fabbri et al. [51]. Main peaks present from the analysis of the linen thread 910.63.7 after it was extracted using hot water (Figure 7d) include the saccharide marker (14), the cellulose marker (15), and Schellmannose (16). This combined EIC for thread 910.63.7 may be compared directly to that of Figure 5f, which shows the thread prior to extraction. In Figure 7d there are no longer any peaks present from a soluble plant gum. However, Schellmannose (16) is still present. This is a reminder that the pyrolysate forms from both water-soluble starch and water-insoluble cellulose.

The TMAH Py-GC-MS analysis of the hot water extracts provided more evidence about possible carbohydrate coatings on the threads than the analysis of the whole threads. However, due to the high alkalinity of the TMAH reagent and thermolysis reactions that can occur during carbohydrate analysis such as racemisation, reduction, and decarboxylation, the technique does not always provide enough information to discern between polysaccharides [67]. For instance, in this case, the reagent cannot differentiate between arabinose and xylose in a polysaccharide chain. This is because these sugars are C2 epimers that form the same deoxy-pentonic acid marker compound upon pyrolysis and methylation [58]. Further investigation into the soluble carbohydrates present in the hot water extracts was undertaken through Py-GC-MS analysis using hexamethyldisilazane (HMDS) derivatisation. This reagent is milder and less alkaline than TMAH; therefore, different pyrolytic pathways and reactions occur through its use [68,69,70]. As a complement to information gained through TMAH Py-GC-MS, the marker compounds that are produced can help differentiate polysaccharides. Using HMDS pyrolysis, the *m*/*z* 217 ion fragment is characteristic in the mass spectra of trimethylsilylated (TMS) saccharide compounds [71,72,73], and therefore, diagnostic for polysaccharide materials. However, because the abundance of this ion in the individual mass spectra varies between marker compounds, the size of peaks shown in EICs do not necessarily indicate their actual relative abundances. Nevertheless, it is a useful tool for investigating polysaccharides [71,72,73].

**Figure 8:**
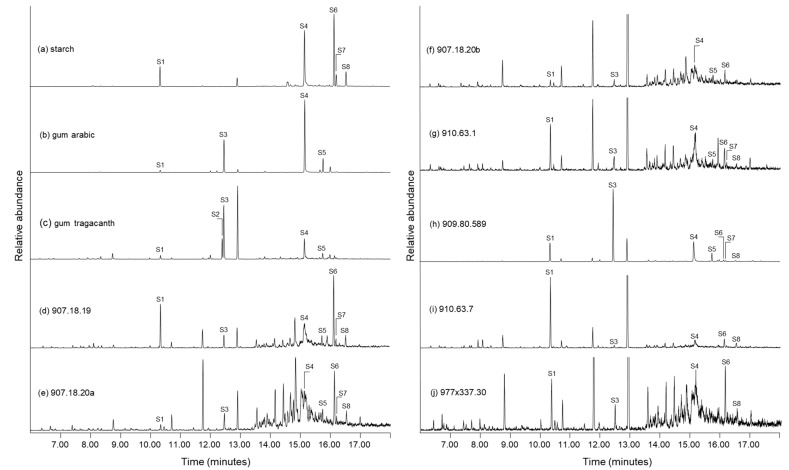
Extracted ion chromatograms (*m*/*z* 217) from the HMDS Py-GC-MS analysis of hot water extracts of reference materials (starch, gum arabic, and gum tragacanth) and linen threads from pleated textiles. Labels correspond to compounds presented in Table 2.

In Figure 8, the EICs (*m*/*z* 217) are shown for the HMDS Py-GC-MS analyses of the hot water extracts from all seven pleated cloths, as well as similar extracts from reference starch, and commercial gum arabic and gum tragacanth. Compounds corresponding to the peak labels are presented in Table 2. There are eight marker compounds present in the EICs, and some, like S1 (unidentified) and S4 (diTMS derivative of levoglucosan), are not as diagnostic because they are common to many polysaccharide sources; these compounds are present in each of the reference materials and the thread extracts in Figure 8. However, other derivatised saccharide pyrolysates are specific to plant gums, including fully derivatised anhydro-fucopyranose (S2), arabinofuranose (S3), and anhydro-galactopyranose (S5). Arabinogalactan polysaccharides, such as gum arabic and many fruit gums, are characterised by predominant abundances of both arabinose and galactose units in the main polymer chain. Alternatively, gum tragacanth is distinguished from arabinogalactans through the presence of a significant relative abundance of fucose and a much lower relative abundance of galactose in the saccharide polymer [58,63,64,65].

Through these complementary analyses, it is evident that the deoxy-pentonic acids (10, 12) formed in the TMAH pyrolyses of the thread extracts (Figure 6) were derived from arabinose and not xylose. All of the thread extract chromatograms in Figure 8 show the presence of fully derivatised arabinofuranose (S3). Other plant gum markers present in the EICs include anhydro-galactopyranose (S5) and anhydro-fucopyranose (S2). The latter fucose marker was only detected in the gum tragacanth reference, and its absence from the thread extracts probably indicates that gum tragacanth was not applied to any of the cloths. However, in the extracts of 907.18.20b and 910.63.7, the arabinofuranose peak is weakly abundant, and since the fucose marker, if present, would only be roughly one third the size of this peak, it is possible that fucose could be present in these chromatograms, and not showing as a resolved peak. Yet it is interesting to note that even on more modern 19th-century textiles, gum tragacanth was rarely used as a size or coating [77]. The gum has poor solubility in water, forms thick, ropey solutions, and even when strained can leave particles on the textile surfaces [77]. Contrary to the missing fucose derivative, the galactose marker compound (S5) was identified in the gum arabic reference, the gum tragacanth reference, and in five of the thread extracts (907.18.19, 907.18.20a, 907.18.20b, 910.63.1, and 909.80.589).

Starch is a glucose-based polysaccharide made up of glucose units in α-D-(1–4) linkages and α-D-(1–6) linkages [78]. Marker HMDS pyrolysates formed through analysis of the reference starch extract (Figure 5a) include fully derivatised levoglucosan (S6), anhydro-glucopyranose (S7) and anhydro-glucofuranose (S8). Although markers S6–S8 also form through similar analysis of dextrin [72] and cellulose [68], these substances are not likely the source of the compounds in the sample extracts. Dextrin is a water-soluble substance that was first made industrially in the 19th century through pyro-processing starch and is probably not present on the ancient cloths [79], and through careful sampling of the extracts using a stereomicroscope and surgical tools, no cellulose fibres were present in the analysed hot water extracts from the cloths. Of the three markers, S6 and S8 are the most relatively abundant in starch and the S7 peak is much smaller in the EIC. Yet, all three peaks were identified in extracts of cloths 907.18.19, 907.18.20a, 910.63.1, and 909.80.589. In the remaining three extracts from cloths 907.18.20b, 910.3.7, and 977 × 337.30, the two most abundant compounds (S6 and S8) of the markers were identified.

The full analytical results for the investigation are presented in Table 3. Based on the results from both sets of Py-GC-MS analyses of the hot water extracts, using TMAH and HMDS, it seems likely that each of the pleated cloths had been treated with a plant gum and a water-soluble polyglucoside, possibly starch. In terms of the plant gum identification, the presence of arabinose and probable absence of fucose may indicate that these threads were all treated with an arabinogalactan gum, such as gum arabic or cherry gum, which have similar carbohydrate profiles [59,72,73]. Fruit gums, however, generally have poor solubility in water, are dark in colour when compared to gum arabic [59], and they may not have been suitable for use as a cloth sizing material. Although Py-GC-MS can provide useful information on the presence of many saccharide constituents, it remains challenging to assign absolute identity to traces of ancient and degraded polysaccharides. This is especially true when there is significant variability in the relative proportion of polysaccharide constituents in plant gums, even within the same genus or species [59,64,65,80], and when mixtures of different polysaccharide materials are likely present [59,63]. This means that although it seems probable that plant gum was applied to the linens, either in spinning, consolidating, and strengthening the warp threads, or sizing the cloth to hold pleats, a cautious approach to the identification of traces of degraded and aged polysaccharides from this ancient period is warranted. Therefore, from these results we conclude only that the cloths, those that had been coated with embalming resins and those without, were each treated with solutions of plant gum and possibly starch.

These two polysaccharide substances may have been applied to the cloths for the purpose of holding the intricate pleat patterns. It has been postulated that a stiffening agent was used to set the pleats in Pharaonic linen clothing [3,5,6,8]. Thus, whether folded by hand or produced using a pleating board [3,5,7,8], it is very likely that the pleats in these cloths have been reinforced and stabilised through the use of applied solutions of starch and plant gum. The question of when these substances were applied remains. It is possible that the gum and the starch were applied at different stages in the production of the cloths. For instance, one substance may have been applied as an agglutinant to consolidate and strengthen the warp threads prior to weaving, and a second application may have occurred after the cloth was woven, prior to being pleated. Another question is whether the plant gum and starch were used on these cloths because they were designed to be pleated, or if these sizings were used universally on woven linen in Ancient Egypt to produce linen cloth that could be useful for multiple purposes, be they ritual, ceremonial, or quotidian.

## 3. Materials and Methods

### 3.1. Reference Materials

Reference materials used in this study include commercial gum arabic and gum tragacanth (Sigma Chemical Company, St, Louis, MO, USA) and precipitated wheat starch (Zin Shofu, Polistini Conservation Material, Washington DC, USA).

### 3.2. Archaeological Samples

Samples measuring between 5–10 mm (w) × 5–10 mm (l) were collected from seven Ancient Egyptian pleated linen cloths from the collection of the ROM. In most instances these were small fragments that had broken off from the main areas of the cloth. Details and photographs of the objects from which the samples were removed may be found in Table 4. Four of the cloths were excavated by Currelly in 1906 from the tomb of King Nebhepetre, now known as Mentuhotep II, (Dynasty XI) in Deir el-Bahri and were accessioned by the museum in 1907 (907.18.19, 907.18.20a, and 907.18.20b) and 1910 (910.63.1). Little provenance is known for the remaining three pleated textile fragments, with the exception that they were removed from excavations in Egypt. Two were accessioned by the museum in 1909 (909.80.589) and 1910 (910.61.7), and the third was accessioned in 1977 (977 × 337.30). All three were obtained by Currelly in Egypt while he was participating in the 1906 excavation.

### 3.3. Scanning Electron Microscopy-Energy Dispersive X-ray Spectrometry

SEM/EDS analysis was performed on the whole threads using a Hitachi S-3500 N VP SEM integrated with an Oxford Inca X-act analytical silicon drift X-ray detector and an AZtec X-ray microanalysis system. The SEM was operated at an accelerating voltage of 20 kV at a pressure of 60 Pa using a backscattered electron detector. With this technique, elemental analysis of volumes down to a few cubic micrometers can be obtained for elements from boron (B) to uranium (U) in the periodic table at a level of approximately 0.1–1% or greater.

### 3.4. Polarised Light Microscopy

For PLM, the fibres were prepared as dispersions in Cargille Meltmount mounting medium (n = 1.66) and examined using a Leica DMRX polarizing light microscope. Lugol’s stain (potassium iodide/iodine, 0.1 N) was employed on the threads and dried hot water extracts were used to test for the presence of starch.

### 3.5. Stereomicroscopy

Small woven fragments from the cloths, individual threads, and dried hot water extracts were examined and photographed using a Leica M205C stereomicroscope interfaced to a DMC 5400 digital camera. All images were collected under normal light illumination, and the small woven fragments were also photographed under UV illumination using a Labino UVG3 2.0 Spotlight. Image processing was undertaken using Leica LASX software. Some images were taken using the LASX automatic z-stacking function, which creates a high-quality extended depth of field image with system optimized acquisition increments calculated by the software.

### 3.6. Gas Chromatography-Mass Spectrometry

For each sample, a fine thread of approximately 3–5 mm in length was placed into a 2 mL clear glass GC–MS vial (Agilent Technologies). To each of the vials 10 μL of *m*-(trifluoromethyl)phenyltrimethylammonium hydroxide (TMTFTH, 0.2 N in methanol)(TCI America, Portland, OR) and 10 μL of toluene were added. The vials were capped with PTFE/silicon/PTFE septa screw top vials (Agilent Technologies, Palo Alto, CA) and placed in a block heater at 60 °C. After 1 h the vials were removed from the heater and centrifuged at 1500 rpm for 1 min. For each analysis, 2 μL of an extract was injected into a glass micro-vial (Agilent Technologies) set in the thermal separation probe (TSP, Agilent Technologies). The TSP was then inserted into a multimode inlet on an Agilent 7890 A GC interfaced to a 5975 C MS. During analysis the inlet temperature was ramped from 50 °C to 250 °C, at a rate of 900 °C /min and held for approximately 38–40 min. Then, at this point in each run the inlet was cleaned by heating to 450 °C, at a rate of 900 °C /min, and held for 3 min before cooling once again to 250 °C. This built-in pyrolytic cleaning cycle at the end of each run helps to mitigate any sample carry-over from the inlet and produces a chromatographic feature that appears to be a short rise and fall in the baseline and can be seen in Figure 4b,c. For the GC separation, a Phenomenex ZB-5MSi fused silica column (30 m × 0.25 mm i.d., 0.25 μm film thickness with 5 m guard column; Phenomenex Inc., Torrance, CA, USA) was used. Ultra-high purity helium carrier gas was used with a constant flow of 1.2 mL/min. The oven was programmed from 40 °C to 200 °C (at 10 °C/min), and then from 200 °C to 310 °C (at 6 °C/min) with a final hold time of 20 min (54.33 min run time). A solvent delay of 10 min was employed. The MS transfer line temperature was held at 280 °C; the MS ion source was 230 °C and the MS quadrupole was 150 °C. The MS was run in scan mode from 45–550 amu (TMAH) (10–25 min), 50–750 amu (25–30 min) and 50–800 amu (30 min–end of run). Agilent ChemStation software, v.E.02.02.2.5 and AMDIS v. 2.71 software were used for data processing.

### 3.7. Pyrolysis-Gas Chromatography-Mass Spectrometry

#### 3.7.1. Whole Threads and Hot Water Extractions

Py-GC-MS analysis was performed both on samples of whole threads from the pleated cloths and on the hot aqueous extracts of the threads. For the whole threads a few linen fibres (approximately 2–3 mm in length) were tweezed from a thread and placed in a glass micro-vial in the thermal separation probe (TSP, Agilent Technologies, Inc., Palo Alto, CA). The samples were then each derivatized using 2 μL tetramethylammonium hydroxide (TMAH, Supelco, Bellafonte, PA, USA) (2.5% in methanol) and analysed in split mode (10:1 or 15:1 split depending on sample size).

For the hot water extractions, approximately 1 cm in total length of threads from each cloth was placed in 100 μL of deionised water in a glass GC vial. The vials were capped and heated for 1 h at 75 °C, then centrifuged for 1 min at 1500 rpm. The water extracts were carefully pipetted into the wells of a spot plate and allowed to dry, then the dried surfaces were gently brushed with a fine sable hair paint brush to remove extraneous fibres. The extracts were sampled under a stereomicroscope using the tip of a scalpel. Scrapings of a few micrograms in total were required for each analysis. The scrapings were placed in glass micro-vials and run in splitless mode once using TMAH derivatisation (2 μL) and once using hexamethyldisilazane (HMDS, Supelco, Bellafonte, PA, USA) (2 μL) derivatisation.

#### 3.7.2. Instrumental Conditions

Pyrolysis was carried out using Direct Inlet pyrolysis-gas chromatography-mass spectrometry (DIP-GC-MS) [81]. For each analysis, a solid sample (either whole thread fragments or hot water extraction residues) was placed in a glass micro-vial in a TSP with derivatizing reagent. The probe was then inserted into a multimode inlet on an Agilent 7890A GC interfaced to a 5975C MS. The inlet temperature was ramped from 50 °C to 450 °C, at a rate of 900 °C/min. The final temperature was held constant for three minutes and then decreased to 250 °C at a rate of 50 °C/min and held for the duration of the run. For the GC separation, a Phenomenex ZB-5MSi fused silica column (30 m × 0.25 mm i.d., 0.25 μm film thickness with 5 m guard column; Phenomenex Inc., Torrance, CA, USA) was used. Ultra-high purity helium carrier gas was used with a constant flow of 1.2 mL/min. The oven was programmed from 40 °C to 200 °C (at 10 °C/min), and then from 200 °C to 310°C (at 6 °C/ min) with a final hold time of 20 min (54.33 min run time). A solvent delay of 5.1 min was employed. The MS transfer line temperature was held at 280 °C; the MS ion source was 230 °C and the MS quadrupole was 150 °C. The MS was operated in electron impact (EI) positive ion mode (70 eV). Scans were run from 45–550 amu (5–25 min), 50–750 amu (25–30 min) and 50–800 amu (30 min–end run). Agilent ChemStation software, v.E.02.02.2.5 and AMDIS v. 2.71 software were used for data processing.

## 4. Conclusions

Over one hundred years has passed since the accessioning of a group of linen fragments into the Egyptian collections of the ROM, acquired by Charles T. Currelly, most likely during his excavations at Deir el-Bahri’s temple and tomb complex. The analysis of seven of these pleated linen cloths rediscovered in the ROM’s Egyptian collections by a combined methodology identified mixtures of organic materials indicative of daily and ritual life in Ancient Egypt. Three were coated with embalming mixtures containing *Pinaceae* resin, mastic resin, and a conifer oil likely made primarily from a juniper species tree or shrub. It is conceivable that these three cloth fragments were cut from clothing and re-purposed as bandages for mummified remains. Apart from the embalming resins, the cloths are remarkably similar both in appearance and composition. All were made in an uneven weave resulting in either warp-faced or weft-faced finished goods. The presence of traces of tannins and humic substances on the threads was so consistent in relative abundance on the seven cloths that we postulate that these substances may result from a common processing treatment, such as retting the flax plants in slow moving water.

Threads from each of the cloth fragments contained traces of gelatinised starch and plant gum, confirming suspicions from past and current scholars that these types of cloths were treated with a substance to reinforce the pleats and lightly stiffen the textiles. Through multiple complementary Py-GC-MS analyses, mixed carbohydrate coatings were distinguished from the carbohydrate linen substrates based on the identification of pyrolysate markers arising from both TMAH and HMDS derivatisation. The relative complexities in identifying the use of starch and plant gum to coat and/or size linen cloth provides a unique insight into the lives of Ancient Egyptians, and a glimpse into a cyclical textile economy of linen consolidation, weaving, fabric preparation, textile use and re-use.

The ROM’s Egyptian collections hold other textile treasures acquired by Currelly during his excavations in Deir el-Bahri, including non-pleated fragments of linen cloth and painted linen votive offerings. Based on the findings of this present study, future work with these textiles will include determining whether they too contain polysaccharide applications. This may provide more information on not only the processing and manufacture of these fine cloths, but also their use in ritual aspects of Ancient Egyptian life and after-life.

## Figures and Tables

**Table 1:** List of compounds corresponding to the labelled peaks in Figure 3, Figure 4, Figure 5, Figure 6 and Figure 7.

Label	Compound	MW (% Rel Abund)	Characteristic Fragment Ions *m*/*z* (% Rel Abundance)	RI	Source
1	2(5H)-furanone	84 (40)	55 (100), 54 (22), 39 (17), 37 (6), 53 (4)	921	c,s,
2	2-hydroxy-3-methyl-2-cyclopenten-1-one	112 (100)	55 (48), 69 (47), 83 (30) 97 (6)	1029	c,s,
3	3-ethyl-2-hydroxy-2-cyclopenten-1-one	126 (100)	55 (39), 83 (38) 69 (32), 97 (18), 111 (16)	1075	c,s,
4	2-methoxy-phenol	124 (68)	109 (100), 81 (67), 53 (22)	1092	c,s,
5	unidentified	126 (1)	57 (100), 58 (10), 71 (4), 87 (2), 98 (<1)	1097	c,s,
6	dihydroxy-methoxy-benzene	140 (100)	69 (63), 97 (48) 125 (31)	1153	c,s
7	dimethyl-methoxy-3(2H)-furanone (tentative)	142 (100)	57 (38), 71 (29), 127 (28), 82 (28), 113 (23)	1220	c,s,
8	unidentified	156 (100)	57 (35), 141 (27), 95 (27), 127 (22)	1237	c,s,
9	dimethoxy phenol (isomer)	154 (100)	53 (40), 139 (33), 83 (28), 125 (27), 111 (22)	1243	c,s,
10	tri-*O*-methyl-3-deoxy-D-*threo*-pentonic acid, methyl ester (ARA/XYL 1) ^a^	206 (<1)	129 (100), 75 (35), 115 (35), 161 (24), 101 (19)	1282	g,
11	dimethoxy phenol (isomer)	154 (100)	139 (79), 53 (46), 66 (29), 125 (27), 83 (27)	1289	c,s,
12	tri-*O*-methyl-3-deoxy-D-*erythro*-pentonic, methyl ester (ARA/XYL 2) ^a^	206 (<1)	129 (100), 115 (37), 75 (37), 101 (24), 161 (24)	1300	g,
13	dimethoxy phenol (isomer)	154 (100)	139 (90), 111 (54), 96 (13), 53 (10)	1334	c,s,
14	1,2,4-trimethoxy benzene	168 (96)	153 (100), 125 (79), 110 (34), 69 (20)	1366	c,s,g
15	unidentified carbohydrate (cellulose, poss. glucopyranoside)		88 (100), 87 (67), 73 (48), 103 (29)	1375	c
16	unidentified carbohydrate (Schellmannose)	204	101 (100), 99 (33), 71 (26), 127 (30), 88 (21), 159 (3)	1385	c,s
17	α-cedrene	204 (47)	119 (100), 93 (41), 91 (31), 105 (30), 161 (23)	1435	j
18	β-cedrene	204 (48)	161 (100), 69 (60), 93 (44), 120 (31), 133 (26)	1444	j
19	2,4,5,6-tetra-*O*-methyl-3-deoxy-D-*xylo*-hexonic acid, methyl ester (GAL 1) ^a^	250 (<1)	129 (100), 75 (30), 101 (27), 161 (21), 191 (8)	1474	g
20	1,6-anhydro-β-D-glucopyranose (levoglucosan)	162 (<1)	60 (100), 43 (81), 57 (3), 73 (60), 98 (8)	1479	c,s,
21	2,4,5,6-tetra-*O*-methyl 3-deoxy-D-*lyxo*-hexonic acid, methyl ester (GAL 2) ^a^	250 (<1)	129 (100), 75 (33), 101 (27), 161 (20), 191 (5)	1499	g
22	cuparene	202 (20)	132 (100), 145 (28), 119 (26), 105 (18)	1527	j
23	cedrol	222 (2)	95 (100), 150 (69), 151 (68), 81 (52), 207 (19)	1616	j
24	calamenene	202 (20)	159 (100), 160 (13), 144 (6), 129 (6)	1693	j
25	cadalene	198 (46)	183 (100), 168 (18), 153 (15), 165 (15), 141 (8)	1702	j
26	3,4,5-tri-*O*-methyl gallic acid, methyl ester	226 (100)	211 (44), 195 (28), 155 (27), 183 (10)	1731	t
*	3,6-dimethoxy-1,2,4,5-tetrachlorobenzene	274 (54)	261 (100), 259 (79), 276 (68), 209 (39), 211 (37), 87 (41)	1745	
27	1,2,3-benezenetricarboxylic acid, trimethyl ester ^b^-	252 (<1)	221 (100), 236 (18)	1833	t
28	unidentifed (tannin)		224 (100), 255 (28), 194 (25), 165 (17), 137 (10)	1842	t
29	1,2,4-benzenetricarboxylici acid, trimethyl ester^b^	252 (4)	221 (100), 103 (10), 193 (8)	1851	t
30	hexadecanoic acid, methyl ester	270 (15)	74 (100), 87 (74), 143 (29), 241 (23)	1930	f
31	4,5-dimethoxy-1,2-benzenedicarboxylic acid, dimethyl ester ^b^	254 (52)	223 (100), 122 (6), 152 (4)	1937	t
32	3-methoxy-1,2,4-benezenetricarboxylic acid, trimethyl ester ^b^	282 (12)	251 (100), 219 (15), 192(8), 134 (4)	2052	t
33	octadecanoic acid, methyl ester	298 (22)	74 (100), 87 963), 143 (17), 255 (8), 199 (6)	2118	f
34	1,2,3,4-benzenetetracarboxylic acid, tetramethyl ester ^b,c^	310 (3)	279 (100), 104 (9), 162 (6), 233 (3)	2135	t
35	1,2,4,5-benzenetetracarboxylic acid, tetramethyl ester ^b,c^	310 (4)	279 (100), 162 (11), 75 (10), 177 (5), 251 (5)	2161	t
36	1,2,3,5-benzenetetracarboxylic acid, tetramethyl ester ^b,c^	310 (<1)	279 (100), 162 (4), 220 (2), 103 (2), 75 (2)	2198	t
37	retene	234 (70)	219 (100), 204 (34), 189 (25)	2229	p
38	2-methoxy-1,3,4,5-tetracarboxylic acid, tetramethyl ester ^b^	340 (10)	309 (100), 277 (5), 134 (4), 191 (4), 263 (4)	2331	t
39	dehydroabietic acid, methyl ester	314 (17)	239 (100), 299 (20), 314 (17), 141 (7)	2350	p
40	7-methoxy-tetradehydroabietic acid, methyl ester	342 (100)	267 (89), 227 (55), 283 (43)	2460	p
41	7-methyoxy-6,8,11,13,15-pentadehydroabietic acid, methyl ester ^c^	340 (100)	265 (8), 225 (56), 281 (38)	2558	p
42	7-oxo-dehydroabietic acid, methyl ester	328 (34)	253 (100), 187 (23), 213 (11), 269 (11)	2589	p
43	7,15-dimethoxy-tetradehydroabietic acid, methyl ester	372 (78)	297 (100), 340 (60), 141 (57), 313 (56), 357 (47)	2608	p
44	15-hydroxy-7-methoxy-tetradehydroabietic acid	358 (50)	340 (100), 283 (93), 225 (90), 265 (55), 299 (27)	2640	p
45	7-oxo-tetradehydroabietic acid, methyl ester	326 (34)	251 (100), 185 (21), 211 (12)	2673	p
46	15-methoxy-7-oxo-dehyrdoabietic acid	358 (2)	343 (100), 344 (22), 327 (6), 283 (4)	2748	p
47	15-hydroxy-7-oxo-dehydroabietic acid, methyl ester	344 (3)	329 (100), 269 (15), 128 (15)	2773	p
48	unidentified triterpenoid	454 (7)	189 (100), 439 (24), 119 (24), 203 (17), 249 (16)	3291	m
49	unidentified triterpenoid	500 (25)	189 (100), 119 (40), 203 (37), 81 (32), 262 (28)	3376	m
50	unidentified triterpenoid	500 (11)	203 (100), 262 (60), 189 (36), 143 (25), 81 (20)	3404	m
51	3-oxo-olean-18-en-28-oic acid, methyl ester	468 (12)	189 (100), 203 (26), 249 (21), 119 (19), 409 (9)	3567	m

^a^ [57]; ^b^ [27]; ^c^ [33]; c = cellulose, f = fat or oil, g = gum, j = juniper oil, m = mastic (*Pistacia* sp.) resin, p = *Pinaceae* resin, s = starch, t = tannins and humic substances.

**Table 2:** List of compounds corresponding to the labelled peaks in Figure 8.

Label	Compound	MW (% Rel Abund)	Characteristic Fragment Ions *m*/*z* (% Rel Abundance)	RI	Source ^d^
S1	unidentified		73 (100), 217 (64), 146 (8), 232 (3)	1252	a,t,s
S2	tetra-O-TMS-1,4-anhydrofucopyranose ^a^		73 (100), 217 (41), 147 (16), 268 (9), 244 (5), 191 (3)	1403	t
S3	1,2,3,5-tetra-O-TMS-arabinofuranose ^b^	438 (<1)	73 (100), 217 (48), 147 (33), 129 (15), 230 (12)	1407	a,t
S4	2,4-di-O-TMS-1,6-anhydro-β-D-glucopyranose (levoglucosan) ^c^	380 (<1)	217 (100), 73 (74), 129 (28), 116 (19), 101 (11)	1628	a,t,s
S5	tri-O-TMS-1,4-anhydro-D-galactopyranose ^d^	332 (6)	73 (100), 217 (44), 157 (38), 191 (37), 147 (19), 243 (8), 204 (6)	1682	a,t
S6	tri-O-TMS-1,6-anhydro-β-D-glucopyranose (levoglucosan) ^c^	378 (<1)	73 (100), 204 (71), 217 (52), 147 (27), 129 (15), 333 (15), 103 (9), 243 (3)	1716	s
S7	tri-O-TMS-1,4-anhydro-D-glucopyranose ^d^	332 (5)	73 (100), 217 (49), 191 (34), 157 (27), 147 (20), 129 (12), 204 (10), 243 (6)	1723	s
S8	tri-O-TMS-1,6-anhydro-β-D-glucofuranose ^d^	378 (<1)	217 (100), 73 (74), 116 (13), 101 (10), 129 (9), 319 (8), 157 (5), 191 (5), 243 (3)	1751	s

^a^ [74]; ^b^ [75]; ^c^ [71]; ^d^ [76]; ^d^ a = gum arabic, s = starch, t = gum tragacanth.

**Table 3:** Analytical results.

Textile	Elements ^1^	Identified Components	Original Material
907.18.19	**carbon**, oxygen, (silicon, calcium)	furans, cyclopentenones, etc.	cellulose
		benzenecarboxylic acids	tannins, humic substances
		polysaccharide, containing arabinose and galactose units	plant gum, such as gum arabic or fruit gum
		water-soluble polyglucosides	possible starch
		2,3,5,6-tetrachlroquinonel	pentachloropheno
907.18.20a	**carbon**, oxygen, (silicon, calcium)	furans, cyclopentenones, etc.	cellulose
		benzenecarboxylic acids	tannins, humic substances
		polysaccharide, containing arabinose and galactose units	plant gum such as gum arabic or fruit gum
		water-soluble polyglucosides	possible starch
		oxidized abietanes	*Pinaceae* resin (heated)
		olealane, lupane, and ursane triterpenoids, including moronic acid	*Pistacia* sp. resin (mastic)
		sesquiterpenes: α-cedrene, β-cedrene cedrol, cuparene, calamenene, cadalene	conifer oil, probably juniper oil
		monocarboxylic fatty acids	animal fat or plant oil
		2,3,5,6-tetrachlroquinone	pentachlorophenol
907.18.20b	**carbon**, oxygen, (calcium)	furans, cyclopentenones, etc.	cellulose
		benzenecarboxylic acids	tannins, humic substances
		polysaccharide, containing arabinose and galactose units	plant gum such as gum arabic, fruit gum, or gum tragacanth
		water-soluble polyglucosides	possible starch
		oxidized abietanes	*Pinaceae* resin (heated)
		oleanane, lupane, and ursane triterpenoids, including moronic acid	*Pistacia* sp. resin (mastic)
		sesquiterpenes: α-cedrene, β-cedrene, cedrol, cuparene, calamenene, cadalene	conifer oil, probably juniper oil
		monocarboxylic fatty acids	animal fat or plant oil
		2,3,5,6-tetrachlroquinone	pentachlorophenol
910.63.1	**carbon**, oxygen, (calcium, silicon)	furans, cyclopentenones, etc.	cellulose
		benzenecarboxylic acids	tannins, humic substances
		polysaccharide, containing arabinose and galactose units	plant gum such as gum arabic or fruit gum
		water-soluble polyglucosides	possible starch
		monocarboxylic fatty acids	animal fat or plant oil
		2,3,5,6-tetrachlroquinone	pentachlorophenol
909.80.589	**carbon**, oxygen, (silicon, calcium)	furans, cyclopentenones, etc.	cellulose
		polysaccharide, containing arabinose and galactose units	plant gum such as gum arabic or fruit gum
		water-soluble polyglucosides	possible starch
		monocarboxylic fatty acids	animal fat or plant oil
910.63.7	**carbon**, oxygen (calcium, silicon)	furans, cyclopentenones, etc.	cellulose
		benzenecarboxylic acids	tannins, humic substances
		polysaccharide, containing arabinose and galactose units	plant gum such as gum arabic, fruit gum, or gum tragacanth
		water-soluble polyglucosides	possible starch
		monocarboxylic fatty acids	animal fat or plant oil
		2,3,5,6-tetrachlroquinone	pentachlorophenol
977 × 337.30	**carbon**, oxygen, (calcium, sulfur, sodium, iron)	furans, cyclopentenones, etc.	cellulose
		benzenecarboxylic acids	tannins and humic substances
		polysaccharide, containing arabinose and galactose units	plant gum such as gum arabic or fruit gum
		water-soluble polyglucosides	possible starch
		oxidized abietanes	*Pinaceae* resin (heated)
		oleanane, lupane, and ursane triterpenoids, including moronic acid	*Pistacia* sp. resin (mastic)
		sesquiterpenes: α-cedrene, β-cedrene, cedrol, cuparene, calamenene, cadalene	conifer oil, probably juniper oil
		monocarboxylic fatty acids	animal fat or plant oil
		2,3,5,6-tetrachlroquinone	pentachlorophenol

^1^ Relative abundance: **major**, minor, (trace).

**Table 4:** Objects analysed.

Objects	Details	Overview Photographs ^a^
Pleated textile fragment, linen, 907.18.19 162.6 cm (L), 48.25 cm (W) -Royal Ontario Museum	11th Dynasty Middle Kingdom c. 2055-1986 BCE -excavated at Deir el-Bahri, Egypt	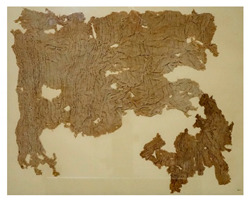
Pleated textile fragment, linen, 907.18.20a 36.5 cm (L), 27.5 cm (W) -Royal Ontario Museum	11th–13th Dynasty Middle Kingdom, c. 2055-1650 BCE -excavated at Deir el-Bahri, Egypt	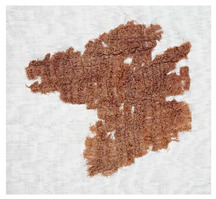
Double-pleated textile fragment, linen, 907.18.20b 36 cm (L), 20.5 cm (W) -Royal Ontario Museum	11th Dynasty Reign of Mentuhotep II Middle Kingdom, c. 2055-2004 BCE -excavated at Deir el-Bahri, Egypt	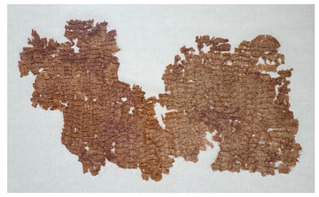
Pleated textile fragment, linen, 910.63.1 5 cm (L), 4 cm (W) -Royal Ontario Museum	11th Dynasty Reign of Mentuhotep II Middle Kingdom, c. 2055-2004 BCE -excavated at Deir el-Bahri, Egypt	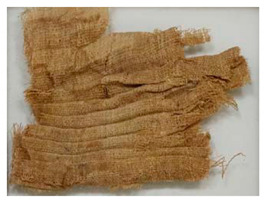
Pleated textile fragment, linen, 909.80.589 19 cm (L), 14.5 cm (W) -Royal Ontario Museum	Egypt	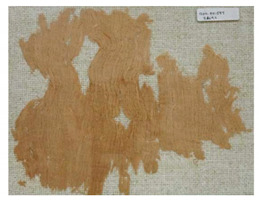
Pleated textile fragment, linen, 910.63.7 6 cm (L), 4 cm (W) -Royal Ontario Museum	Egypt	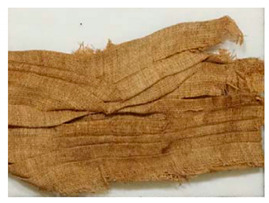
Pleated textile fragment, linen, 977 × 337.30 14.5 cm (L), 10 cm (W) -Royal Ontario Museum	Egypt	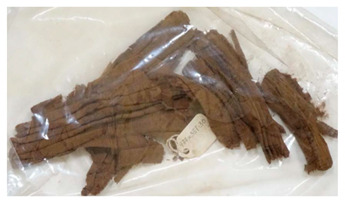

^a^ Copyright Royal Ontario Museum.

## Data Availability

Please contact the authors to obtain copies of data files.

## Supplementary Materials

The following are available online at https://www.mdpi.com/article/10.3390/molecules27134103/s1, Figure S1: Photomicrographs of threads from each cloth before and after staining, and Figure S2: Extracted ion chromatograms highlighting tannin and humic substances present on the cloths.

## References

[1] Currelly C.T. (1956). I Brought the Ages Home.

[2] Naville E., Hall H.R., Currelly C.T. (1913). The XIth Dynasty Temple at Deir El-Bahari, Part III.

[3] Needler W., Gervers V. (1977). Three Pieces of Unpattern Linen from Ancient Egypt in the Royal Ontario Museum. Studies in Textile History.

[4] Currelly C.T. (1905). Letter from C.T. Currelly to Mr. Walker.

[5] Riefstahl E. (1970). A Note on Ancient Fashions Four Early Egyptian Dresses. Boston Mus. Bull..

[6] Riefstahl E., John B. (1944). Patterned Textiles in Pharaonic Egypt.

[7] Vogelsang-Eastwood G., Nicholson P.T., Shaw I. (2000). Textiles. Ancient Egyptian Materials and Technology.

[8] Jones J. (2014). The Enigma of the Pleated Dress: New Insights from Early Dynastic Helwan Reliefs. J. Egypt. Archaeol..

[9] Vogelsang-Eastwood G. (1992). The Production of Linen in Pharaonic Egypt.

[10] Hübner J. (1909). The Analysis of Some Ancient Egyptian Fabrics. J. Soc. Dye. Colour..

[11] Poulin J., Moriarty M. The Identification of Yellow Iron Buff Dye on Egyptian Textiles. In Proceedings of Dyes in History and Archaeology 37.

[12] Edwards H.G., Currie K.J., Ali H.R., Jorge Villar S.E., David A.R., Denton J. (2007). Raman Spectroscopy of Natron: Shedding Light on Ancient Egyptian Mummification. Anal. Bioanal. Chem..

[13] Lewin M. (1997). Oxidation and Aging of Cellulose. Macromol. Symp..

[14] De Caro L., Matricciani E., Fanti G. (2020). Yellowing of Ancient Linen and Its Effects on the Colours of the Holy Face of Manoppello. Heritage.

[15] Mosca Conte A., Pulci O., Knapik A., Bagniuk J., Del Sole R., Lojewska J., Missori M. (2012). Role of Cellulose Oxidation in the Yellowing of Ancient Paper. Phys. Rev. Lett..

[16] Wouters J., Maes L., Germer R. (1990). The Identification of Haematite as a Red Colorant on an Egyptian Textile from the Second Millenium, B.C. Stud. Conserv..

[17] Tamburini D., Dyer J., Vandenbeusch M., Borla M., Angelici D., Aceto M., Oliva C., Facchetti F., Aicardi S., Davit P. (2021). A Multi-Scalar Investigation of the Colouring Materials Used in Textile Wrappings of Egyptian Votive Animal Mummies. Herit. Sci..

[18] Sarret M., Adam P., Schaeffer P., Ebert Q., Perthuison J., Pierrat-Bonnefois G. (2017). Organic Substances from Egyptian Jars of the Early Dynastic Period (3100–2700 BCE): Mode of Preparation, Alteration Processes and Botanical (Re) Assessment of “Cedrium”. J. Archaeol. Sci. Rep..

[19] Barnes K.M., Whiffin A.L., Bulling M.T. (2019). A Preliminary Study on the Antibacterial Activity and Insect Repellent Properties of Embalming Fluids from the 18th Dynasty (1550–1292 BCE) in Ancient Egypt. J. Archaeol. Sci. Rep..

[20] Jones J., Higham T.F., Chivall D., Bianucci R., Kay G.L., Pallen M.J., Oldfield R., Ugliano F., Buckley S.A. (2018). A Prehistoric Egyptian Mummy: Evidence for an ‘Embalming Recipe’and the Evolution of Early Formative Funerary Treatments. J. Archaeol. Sci..

[21] Buckley S.A., Clark K.A., Evershed R.P. (2004). Complex Organic Chemical Balms of Pharaonic Animal Mummies. Nature.

[22] Buckley S.A., Evershed R.P. (2001). Organic Chemistry of Embalming Agents in Pharaonic and Graeco-Roman Mummies. Nature.

[23] Buckley S.A., Stott A.W., Evershed R.P. (1999). Studies of Organic Residues from Ancient Egyptian Mummies Using High Temperature-Gas Chromatography-Mass Spectrometry and Sequential Thermal Desorption-Gas Chromatography-Mass Spectrometry and Pyrolysis-Gas Chromatography-Mass Spectrometry. Analyst.

[24] David R. (2008). Egyptian Mummies and Modern Science.

[25] Poulin J. (2018). A New Methodology for the Characterisation of Natural Dyes on Museum Objects Using Gas Chromatography–Mass Spectrometry. Stud. Conserv..

[26] Khan S.U., Schnitzer M. (1971). Further Investigations on the Chemistry of Fulvic Acid, a Soil Humic Fraction. Can. J. Chem..

[27] del Río J.C., González-Vila F.J., Martín F., Verdejo T. (1994). Characterization of Humic Acids from Low-Rank Coals by 13C-NMR and Pyrolysis-Methylation. Formation of Benzenecarboxylic Acid Moieties during the Coalification Process. Org. Geochem..

[28] Peña-Méndez E.M., Havel J., Patočka J. (2005). Humic Substances-Compounds of Still Unknown Structure: Applications in Agriculture, Industry, Environment, and Biomedicine. J. Appl. Biomed..

[29] Río Andrade J.C., del Hatcher P.G., Gaffney J.S., Marley N.A., Clark S.B. (1996). Structural Characterization of Humic Substances Using Thermochemolysis with Tetramethylammonium Hydroxide. Humic and Fulvic Acids: Isolation, Structure, and Environmental Role.

[30] Poulin J., Moriarty M. (2021). Dye Analysis of Bocksten Man’s Clothing.

[31] Patra A.K., Ramawat K.G., Ahuja M.R. (2016). Linen and Its Wet Processing. Fiber Plants Biology, Biotechnology and Applications.

[32] del Rio J.C., Rencoret J., Gutiérrez A., Nieto L., Jiménez-Barbero J., Martínez Á.T. (2011). Structural Characterization of Guaiacyl-Rich Lignins in Flax (Linum Usitatissimum) Fibers and Shives. J. Agric. Food Chem..

[33] Pastorova I., van der Berg K.J., Boon J.J., Verhoeven J.W. (1997). Analysis of Oxidised Diterpenoid Acids Using Thermally Assisted Methylation with TMAH. J. Anal. Appl. Pyrolysis.

[34] Helwig K., Monahan V., Poulin J. (2008). The Identification of Hafting Adhesive on a Slotted Antler Point from a Southwest Yukon Ice Patch. Am. Antiq..

[35] Hjulström B., Isaksson S., Hennius A. (2006). Organic Geochemical Evidence for Pine Tar Production in Middle Eastern Sweden During the Roman Iron Age. J. Archaeol. Sci..

[36] Modugno F., Ribechini E., Colombini M.P., Ribechini F. (2009). GC/MS in the Characterisation of Resinous Materials. Organic Mass Spectrometry in Art and Archaeology.

[37] Colombini M.P., Modugno F., Silvano F., Onor M. (2000). Characterization of the Balm of an Egyptian Mummy from the Seventh Century, B.C. Stud. Conserv..

[38] Łucejko J.J., Lluveras-Tenorio A., Modugno F., Ribechini E., Colombini M.P. (2012). An Analytical Approach Based on X-Ray Diffraction, Fourier Transform Infrared Spectroscopy and Gas Chromatography/Mass Spectrometry to Characterize Egyptian Embalming Materials. Microchem. J..

[39] Łucejko J., Connan J., Orsini S., Ribechini E., Modugno F. (2017). Chemical Analyses of Egyptian Mummification Balms and Organic Residues from Storage Jars Dated from the Old Kingdom to the Copto-Byzantine Period. J. Archaeol. Sci..

[40] Ménager M., Azémard C., Vieillescazes C. (2014). Study of Egyptian Mummification Balms by FT-IR Spectroscopy and GC-MS. Microchem. J..

[41] Shiojima K., Arai Y., Masuda K., Takase Y., AGETA T., AGETA H. (1992). Mass Spectra of Pentacyclic Triterpenoids. Chem. Pharm. Bull..

[42] van der Doelen G.A., van den Berg K.J., Boon J.J. (1998). Comparative Chromatographic and Mass-Spectrometric Studies of Triterpenoid Varnishes: Fresh Material and Aged Samples from Paintings. Stud. Conserv..

[43] Mills J., White R. (1994). The Organic Chemistry of Museum Objects.

[44] Serpico M., White R., Nicholson P.T., Shaw I. (2000). Resins, Amber and Bitumen. Ancient Egyptian Materials and Technology.

[45] Stern B., Heron C., Corr L., Serpico M., Bourriau J. (2003). Compositional Variations in Aged and Heated *Pistacia* Resin Found in Late Bronze Age Canaanite Amphorae and Bowls from Amarna, Egypt. Archaeometry.

[46] Tabanca N., Nalbantsoy A., Kendra P.E., Demirci F., Demirci B. (2020). Chemical Characterization and Biological Activity of the Mastic Gum Essential Oils of *Pistacia* Lentiscus Var. Chia from Turkey. Molecules.

[47] Koller J., Baumer U., Kaup Y., Schmid M., Weser U. (2003). Analysis of a Pharaonic Embalming Tar. Nature.

[48] Koller J., Baumer U., Kaup Y., Weser U. (2005). Herodotus’and Pliny’s Embalming Materials Identified on Ancient Egyptian Mummies. Archaeometry.

[49] Kremer S., Sterner O., Anke H. (1992). Degradation of Pentachlorophenol by Mycena Avenacea TA 8480-Identification of Initial Dechlorinated Metabolites. Z. Für Nat. C.

[50] Charrié-Duhaut A., Connan J., Rouquette N., Adam P., Barbotin C., de Rozières M.-F., Tchapla A., Albrecht P. (2007). The Canopic Jars of Rameses II: Real Use Revealed by Molecular Study of Organic Residues. J. Archaeol. Sci..

[51] Fabbri D., Helleur R. (1999). Characterization of the Tetramethylammonium Hydroxide Thermochemolysis Products of Carbohydrates. J. Anal. Appl. Pyrolysis.

[52] Schilling M.R., Heginbotham A., van Keulen H., Szelewski M. (2016). Beyond the Basics: A Systematic Approach for Comprehensive Analysis of Organic Materials in Asian Lacquers. Stud. Conserv..

[53] Körber U., Schilling M.R., Dias C.B., Dias L. (2016). Simplified Chinese Lacquer Techniques and *Nanban* Style Decoration on Luso-Asian Objects from the Late Sixteenth or Early Seventeenth Centuries. Stud. Conserv..

[54] Zhao F., Xing H., Wang J., Jia Z., Chao X., Wang J., Liu J., Li Y. (2022). Analytical Investigation of Jiatang Scroll Paintings in the Seventh Year of the Guangxu Era. Coatings.

[55] O’Shea C., Fenn M., Gillis K.Z., Khanjian H., Schilling M. (2021). Korean Lacquerware from the Late Joseon Dynasty: Conservation and Analysis of Four Objects at the Asian Art Museum of San Francisco. Stud. Conserv..

[56] Körber U., Bridgland J. Chinese Lacquer Decorations for Catholic European Consumers in the 16th and 17th Centuries: Stylistic and technical Evolutions. Proceedings of the ICOM-CC 19th Triennial Conference Preprints.

[57] Riedo C., Scalarone D., Chiantore O. (2010). Advances in Identification of Plant Gums in Cultural Heritage by Thermally Assisted Hydrolysis and Methylation. Anal. Bioanal. Chem..

[58] Riedo C., Scalarone D., Chiantore O. (2013). Multivariate Analysis of Pyrolysis-GC/MS Data for Identification of Polysaccharide Binding Media. Anal. Methods.

[59] Lluveras-Tenorio A., Mazurek J., Restivo A., Colombini M.P., Bonaduce I. (2012). Analysis of Plant Gums and Saccharide Materials in Paint Samples: Comparison of GC-MS Analytical Procedures and Databases. Chem. Cent. J..

[60] Scott D.A., Dodd L.S., Furihata J., Tanimoto S., Keeney J., Schilling M.R., Cowan E. (2004). An Ancient Egyptian Cartonnage Broad Collar-Technical Examination of Pigments and Binding Media. Stud. Conserv..

[61] Scott D.A. (2016). A Review of Ancient Egyptian Pigments and Cosmetics. Stud. Conserv..

[62] Scott D.A., Warmlander S., Mazurek J., Quirke S. (2009). Examination of Some Pigments, Grounds and Media from Egyptian Cartonnage Fragments in the Petrie Museum, University College London. J. Archaeol. Sci..

[63] Newman R., Halpine S.M., Davies W.V. (2001). The Binding Media of Ancient Egyptian Painting. Colour and Painting in Ancient Egypt.

[64] Newman R., Serpico M., Nicholson P.T., Shaw I. (2000). Adhesives and Binders. Ancient Egyptian Materials and Technology.

[65] Fulcher K. (2018). Painting Amara West: The Technology and Experience of Colour in New Kingdom Nubia. PhD Thesis.

[66] Twilley J.W., Lambert J.B. (1984). The Analysis of Exudate Plant Gums in Their Artistic Applications: An Interim Report. Archaeological Chemistry-III.

[67] Tamburini D., Bonaduce I., Colombini M.P. (2015). Characterisation of Oriental Lacquers from Rhus Succedanea and Melanorrhoea Usitata Using in Situ Pyrolysis/Silylation-Gas Chromatography Mass Spectrometry. J. Anal. Appl. Pyrolysis.

[68] Fabbri D., Chiavari G. (2001). Analytical Pyrolysis of Carbohydrates in the Presence of Hexamethyldisilazane. Anal. Chim. Acta.

[69] Fabbri D., Prati S., Vassura I., Chiavari G. (2003). Off-Line Pyrolysis/Silylation of Cellulose and Chitin. J. Anal. Appl. Pyrolysis.

[70] Fabbri D., Prati S., Vassura I. (2002). Molecular Characterisation of Organic Material in Air Fine Particles (PM10) Using Conventional and Reactive Pyrolysis-Gas Chromatography-Mass Spectrometry. J. Environ. Monit..

[71] Fabbri D., Chiavari G., Prati S., Vassura I., Vangelista M. (2002). Gas Chromatography/Mass Spectrometric Characterisation of Pyrolysis/Silylation Products of Glucose and Cellulose. Rapid Commun. Mass Spectrom..

[72] Andreotti A., Bonaduce I., Colombini M.P., Modugno F., Ribechini E. (2009). A Diagnosis of the Yellowing of the Marble High Reliefs and the Black Decorations in the Chapel of the Tomb of Saint Anthony (Padua, Italy). Int. J. Mass Spectrom..

[73] Bonaduce I., Andreotti A., Colombini M.P., Ribechini F. (2009). Py-GC/MS of Organic Paint Binders. Organic Mass Spectrometry in Art and Archaeology.

[74] Torri C., Soragni E., Prati S., Fabbri D. (2013). Py-SPME-GC-MS with on-Fiber Derivatization as a New Solvent-Less Technique for the Study of Polar Macromolecules: Application to Natural Gums. Microchem. J..

[75] Tamburini D., Łucejko J.J., Zborowska M., Modugno F., Cantisani E., Mamoňová M., Colombini M.P. (2017). The Short-Term Degradation of Cellulosic Pulp in Lake Water and Peat Soil: A Multi-Analytical Study from the Micro to the Molecular Level. Int. Biodeterior. Biodegrad..

[76] Łucejko J.J., Colombini M.P., Ribechini E. (2020). Chemical Alteration Patterns of Ancient Egyptian Papyri Studied by Pyrolysis-GC/MS with in Situ Silylation. J. Anal. Appl. Pyrolysis.

[77] Ermen W.F.A., Van Nostrand (1912). The Materials Used in Sizing: Their Chemical and Physical Properties, and Simple Methods for Their Technical Analysis and Valuation. A Course of Lectures Delivered at He Manchester School of Technology.

[78] Whistler R.L., Daniel J.R., Whistler R.L., BeMiller J.N., Paschall E.F. (1984). Molecular Structure of Starch. Starch: Chemistry and Technology.

[79] Kennedy H., Fischer Jr A., Whistler R.L., BeMiller J.N., Paschall E.F. (1984). Starch and Dextrins in Prepared Adhesives. Starch: Chemistry and Technology.

[80] Granzotto C., Sutherland K., Arslanoglu J., Ferguson G.A. (2019). Discrimination of Acacia Gums by MALDI-TOF MS: Applications to Micro-Samples from Works of Art. Microchem. J..

[81] Poulin J., Kearney M., Veall M.-A. (2022). Direct Inlet Py-GC-MS Analysis of Cultural Heritage Materials. J. Anal. Appl. Pyrolysis.

