# Supplementary material for: Found in the Folds: A Rediscovery of Ancient Egyptian Pleated Textiles and the Analysis of Carbohydrate Coatings
Source: Molecules. 2022 Jun 25;27(13):4103. doi: 10.3390/molecules27134103 (PMC9268585; doi:10.3390/molecules27134103)
Supplement: Supplementary file 1 [file molecules-27-04103-s001.zip › molecules-1772757-supplementary.pdf]

Found in the Folds: A Rediscovery of Ancient Egyptian Pleated Textiles and the Analysis of Carbohydrate Coatings

Jennifer Poulin [1,*], Chris Paulocik [2] and Margaret-Ashley Veall [1]

[1] Canadian Conservation Institute, 1030 Innes Rd., Ottawa, ON K1B 4S7, Canada; margaret.veall@pch.gc.ca
[2] Royal Ontario Museum, 100 Queen's Park, Toronto, ON M5S 2C6, Canada; chrisp@rom.on.ca
* Correspondence: jennifer.poulin@pch.gc.ca

1. Supporting Information

1.1 Microscopic examination and staining

Threads from each cloth were stained using a solution of potassium iodide/iodine 0.1N (Lugol's stain). Photomicrographs of the threads before and after staining are shown in Figure S1. PLM examination of the cloths did not identify any intact starch granules, therefore, if present a starch coating would be in gelatinised form. Although some of the stained threads in Figure S1 seem to have darkened edges, the lack of continuity and the difficulty in visualizing the staining along the surface, particularly given that a coating would be expected to have a relatively continuous distribution rendered this stream of examination inconclusive.

1.2. TMTFTH extractions: tannins and humic substances

With the exception of cloth 909.80.589, which was a weak extraction, all of the cloths showed the presence of tannins and humic substances in the chromatograms. Extracted ion chromatograms (m/z 221, 223, 224, 226, 251, 279, and 309) for each cloth are presented in Figure S3.

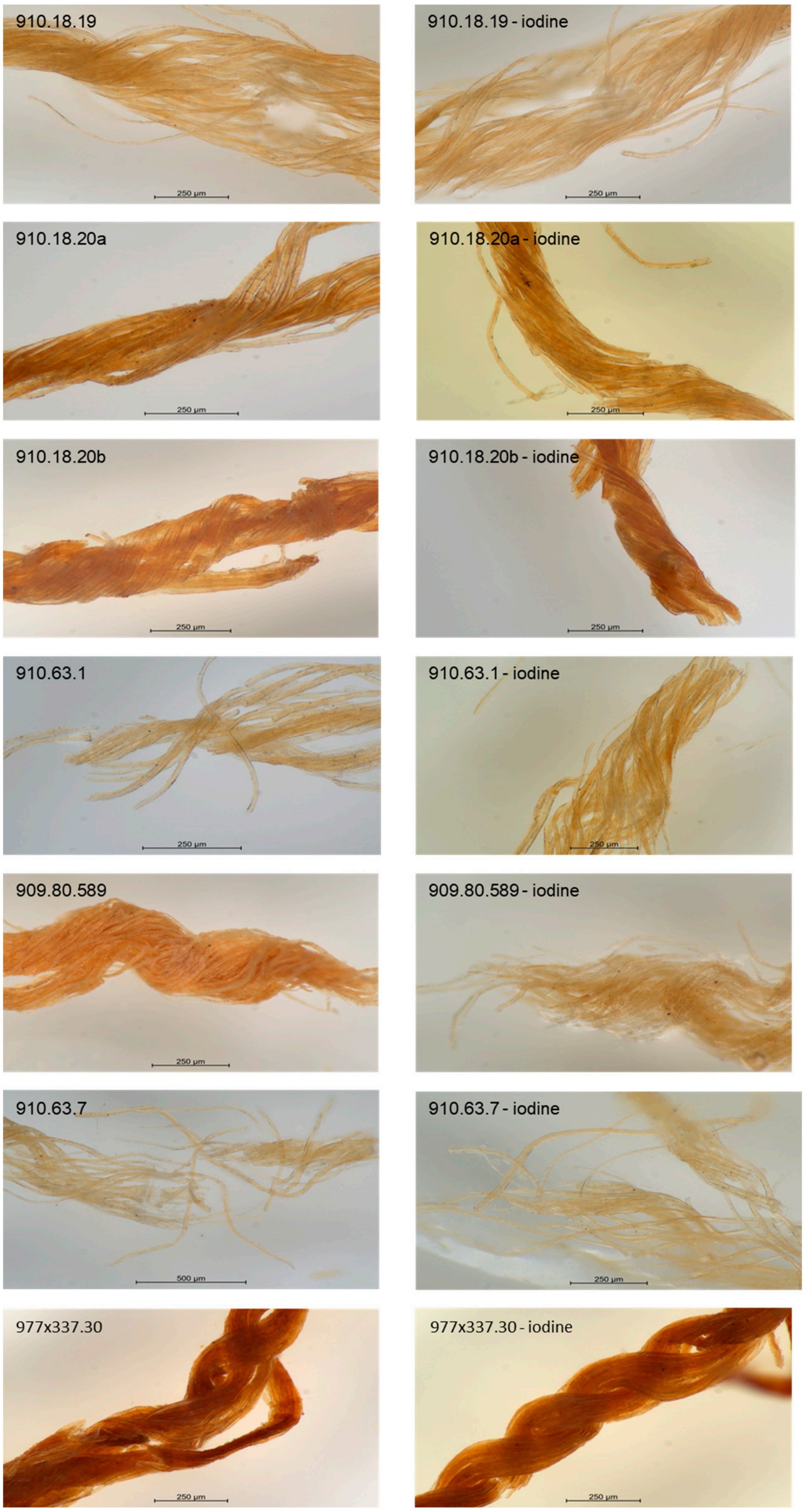

Figure S1. Photomicrographs of threads from each cloth before and after staining with a solution of potassium iodide/iodine 0.1N (Lugol's stain).

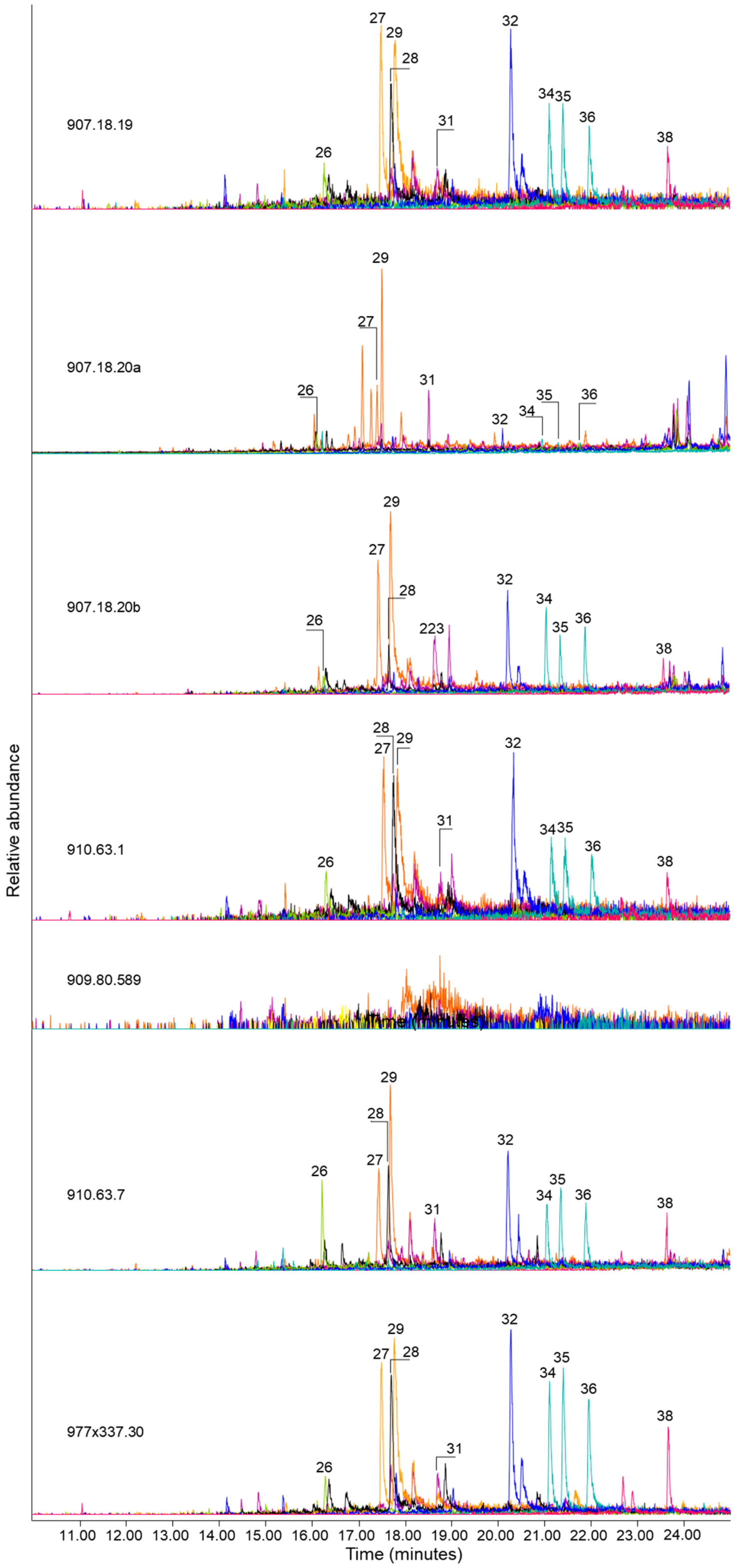

Figure S2. EICs (m/z 221, 223, 224, 226, 251, 279 and 309) of the TMTFTH extract chromatograms for each cloth. Peak labels are described in Table 1.
